# The Influence of Initiator Concentration on Selected Properties on Poly-*N*-Vinylcaprolactam Nanoparticles

**DOI:** 10.3390/nano9111577

**Published:** 2019-11-07

**Authors:** Agnieszka Gola, Aleksandra Niżniowska, Witold Musiał

**Affiliations:** Department of Physical Chemistry, Pharmaceutical Faculty, Wroclaw Medical University, Borowska 211, 50-556 Wroclaw, Poland; agnieszka.gola@umed.wroc.pl (A.G.); aleksandra.nizniowska@gmail.com (A.N.)

**Keywords:** nanoparticles, *N*-vinylcaprolactam, 2-acrylamido-2-methyl-1-propanesulfonic acid, lower critical temperature solution, cationic initiator, conductivity

## Abstract

The thermosensitive polymers of *N*-vinylcaprolactam P1, P2, P3, P4, and P5 were synthesized via the surfactant free precipitation polymerization (SFPP) at 70 °C in the presence of cationic initiator 2,2’-azobis[2-methylpropionamidine] dihydrochloride (AMPA). The influence of various concentrations of initiator AMPA on particle size, aggregation and lower critical temperature solution (LCST) was investigated by dynamic light scattering (DLS) measurement. The conductivity was measured in the course of the synthesis and during temperature decrease of the reaction mixtures. The polymers were characterized by Attenuated Total Reflectance-Fourier Transform Infrared spectroscopy (ATR-FTIR), ^1^H NMR, and thermogravimetric analysis. Thermal parameters of the degradations process were investigated using thermogravimetric analysis (TGA/DTA) under non-isothermal conditions in N_2_ atmosphere. The samples were characterized by powder X-ray diffraction analysis (PXRD).The hydrodynamic diameter (HD), polydispersity index (PDI) and zeta potential (ZP) were measured in aqueous dispersions of the synthesized polymers in temperature 18–45 °C. HD and PDI values at 18 °C were 137.23 ± 67.65 nm (PDI = 0.53 ± 0.18), 83.40 ± 74.46 nm (PDI = 0.35 ± 0.08), 22.11 ± 0.29 nm (PDI = 0.45 ± 0.05), 29.27 ± 0.50 nm (PDI = 0.41 ± 0.04), 39.18 ± 0.57 nm (PDI = 0.38 ± 0.01) for P1, P2, P3, P4, and P5, respectively. The aqueous solutions of the obtained polymers at 18–45 °C had a positive charge. ZP’s for P1, P2, P3, P4, and P5 polymers at 18 °C were 11.64 ± 4.27 mV, 12.71 ± 3.56 mV, 3.24 ± 0.10 mV, 0.77 ± 0.28 mV, 1.78 ± 0.56 mV respectively. The LCST range was between 32 and 38 °C. We conclude that the concentration of initiator affects the size of obtained polymeric spheres and theirs LCST.

## 1. Introduction

Despite continuous advances in pharmacotherapy, there are still many factors that limit effective pharmacological treatment, including drug instability in pH conditions of the gastrointestinal tract, low bioavailability, short half-life time, low drug selectivity, and adverse reactions in healthy cells, e.g., in the treatment of cancer [1]. Thus, many studies are being conducted to eliminate the restrictions of present therapies. Numerous studies evaluate new drug delivery systems for extended control of the pharmacokinetic and pharmacodynamic parameters of medicinal products. Important aspects include the reduction of the side effects, increases in stability, and the development of controlled release mechanisms of the drug under the influence of the pathophysiological factors. These effects may be obtained by using stimuli-sensitive nanocarriers [2,3,4,5,6,7].

Sensitive nanopolymers have significant potential for medical applications due to their specific behavior, depending on the external conditions. The physical and chemical properties are modified as a response to a change in the environmental stimulus, such as temperature, pH, magnetic or electric field, light, mechanical stress, and the presence of a given chemical substance [8,9,10,11]. The sensitive compounds change their properties rapidly at the gentle transition of the critical point of the stimulus; the crucial property of the process is reversibility after discontinuation of the selected factor [12]. Structural modifications may occur within the polymer during transition, covering amphiphilic properties, molecule conformation, phase properties, conductivity, as well as in mechanical properties [4]. Consequently, the polymer may precipitate from the solution, increase or decrease its volume, and release the drug substance. These polymers are, therefore, ideal candidates for the role of "intelligent" drug carriers, i.e., for proteins and nucleic acids.

Temperature-sensitive polymers change their solubility due to temperature fluctuations. Thermosensitive polymers may be applied to hydrogels, capsules, micelles, and nano- and microparticles, interpenetrating polymer networks, fibers, and films [13]. The most widely studied thermally sensitive polymer is poly-*N*-isopropylacrylamide (PNIPAAm), however, it may exert side effects. Another compound—*N*-vinylcaprolactam (NVCL)—presents similar as NIPAAm characteristics, and is contemporarily studied. The *N*-vinylcaprolactam was initially characterized in 1957 [14] and further studied to synthesize poly-NVCL. Poly-NVCL is amphiphilic due to the presence of a hydrophilic lactam ring and a hydrophobic vinyl group in the molecule, which determines the solubility of the compound in both polar and non-polar solvents [15]. In contrast to PNIPAAm, the poly-NVCL is biodegradable and biocompatible. It forms complexes with organic substances and is highly resistant to hydrolysis [16]. The products of the hydrolysis is safe polymeric carboxylic acid, as the amide group in the PNVCL molecule is directly connected to the polyvinyl side chain carbon [17]. Poly-NVCL has a lower critical solubility in the 32–34 °C temperature range, beneficially close to the temperature of the human body [18]. These properties support the role of poly-NVCL as suitable for "smart" drug carriers.

Poly-NVCL is used in hair care products, fluorescent thermometers, and numerous biomaterials. It is also applied for cell immobilization and membrane separation techniques [19]. Poly-NVCL could provide controlled delivery of drugs to a patient’s body.

The aim of this work was the synthesis and characterization of cationic polymers of *N*-vinylcaprolactam P1, P2, P3, P4, and P5, as well as the evaluation of the effect of different concentrations of cationic initiator 2,2’-azobis(2-methylpropionamidine) dihydrochloride (AMPA) on the physicochemical properties of synthesized polymers. The comparison of the properties of the resulting products may enable the determination of initial reaction conditions, which are the most suitable for desirable polymer properties, for use as an intelligent drug carrier operating in response to a change in temperature.

## 2. Results

### 2.1. Synthesis

**Figure 1:**
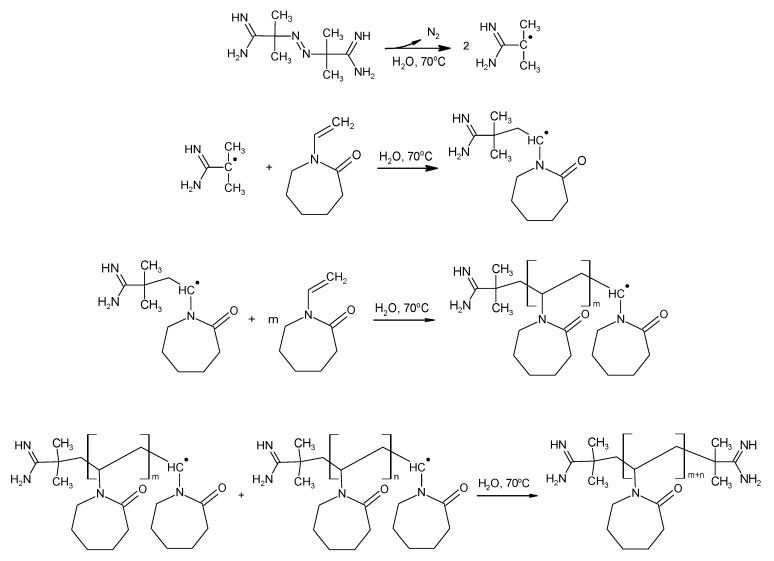
The basic steps of synthesis of PNVCL by polymerization of *N*-vinylcaprolactam (NVCL) aqueous solution in the presence of 2,2’-azobis(2-methylpropionamidine) dihydrochloride (AMPA) initiator.

The present work focuses on the synthesis of five types of polymeric nanoparticles, derivatives of NVCL (P1, P2, P3, P4, and P5), via surfactant-free precipitation polymerization (SFPP), and on the physicochemical study of the synthesized, thermally-responsive systems, both in aqueous solutions and in solid form. The syntheses were carried out under the same physical reaction conditions, including temperature, stirring rate, nitrogen atmosphere, and aqueous solvent, however, the concentrations of cationic initiator 2,2’-azobis[2-methylpropionamidine] dihydrochloride (AMPA) varied. A more detailed description of the synthesis procedure had been reported in Section 4.2, Synthesis. High-temperature conditions of 70 °C led to the decomposition of the initiator molecule and resulted in effective radical formation. The initial molar ratio of monomer-to-radical was maintained at 1.0:1.5, 1.0:1.0, 1.0:0.5, 1.0:0.25, and 1.0:0.125, respectively for P1, P2, P3, P4, and P5 polymers, as presented in Table 1. The introduction of the monomer solution into the reactor resulted in a milky-white dispersion for all tested compositions. The rate of turbidity manifestation over time was related to the initiator concentration in the reactant mixture. Increased concentrations of the initiator accelerated the clouding of the solution. The observed turbidity intensified rapidly and remained constant in the heating process until the end of the synthesis. Reduction of the temperature of the reaction mixture to room temperature resulted in the retrieval of the transmittance. A general diagram of the proposed basic stages of radical polymerization of *N*-vinylcaprolactam is presented in Figure 1.

The aqueous polymer solutions, purified by dialysis method, were freeze-dried. From 100 mL of cleaned solutions of polymers P1, P2, P3, P4, and P5 were obtained about 0.06166, 0.07973, 0.11637, 0.13174 and 0.18569 g of solid product, respectively.

### 2.2. Conductivity Measurements Analysis

**Figure 2:**
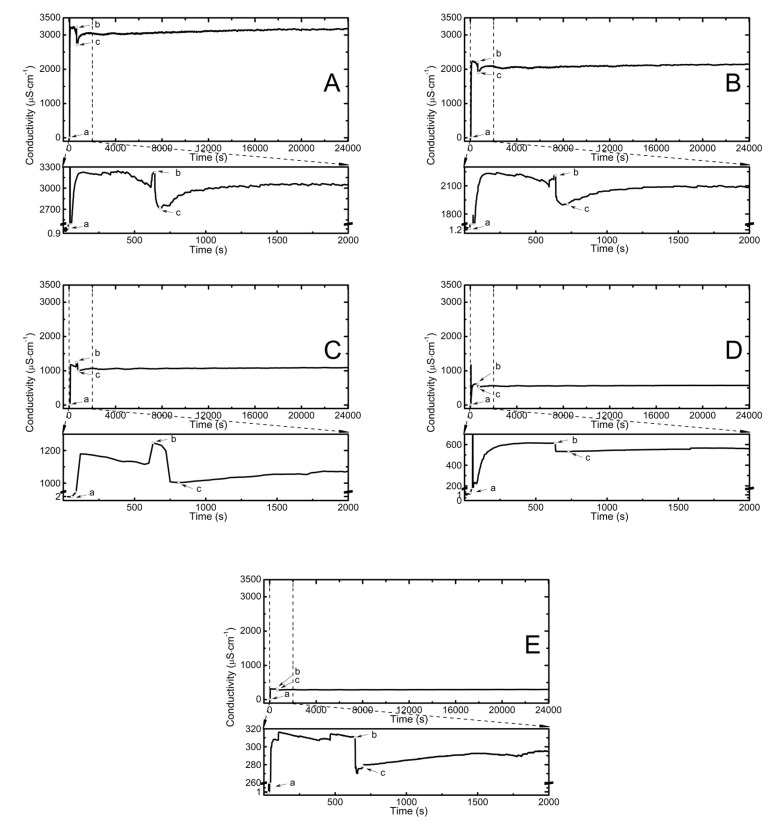
Dependence of conductivity in function of time in reaction mixtures of P1 (**A**), P2 (**B**), P3 (**C**), P4 (**D**), and P5 (**E**) in the course of the polymerization reaction at *T* = 70 °C. Point (a) indicates the addition of an initiator, point (b) indicated the addition of the aqueous solution of the monomer, and point (c) indicated the beginning of turbidity.

The electrolytic conductivity tests were carried out in the course of the polymerization reactions and after synthesis during the cooling of the reaction mixtures. The readings were carried out in one-second time intervals for P1, P2, P4, and P5 and in 30-s intervals for P3. The conductivity profile of aqueous dispersions of P1, P2, P3, P4, and P5 polymers in the course of the polymerization reaction is presented in Figure 2A–E. The initially low conductivity, close to that of deionized water, ca. 0.874 μS cm^−1^, increased rapidly after introducing to the system the respective amount of initiator. The average values were 3200, 2200, 1200, 630, and 320 μS cm^−1^ for P1, P2, P3, P4, and P5, respectively. As expected, the conductivity of the initiator was proportional to its concentration. Addition of the initiator to the reactant mixtures was marked as point (a) in Figure 2A–E. Consequently, after ca. 10 min, addition of aqueous solution of monomer to the reaction system, see point (b) in Figure 2A–E, resulted in an abrupt decrease of the conductivity to ca. 2700, 1900, 1000, 550, and 270 μS cm^−1^ for systems P1, P2, P3, P4, and P5, respectively. The conductivity gradually increased until a plateau phase.

**Figure 3:**
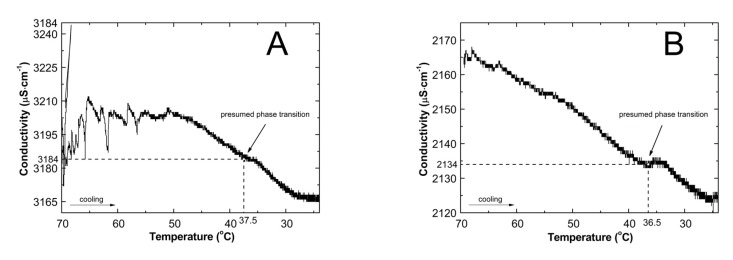

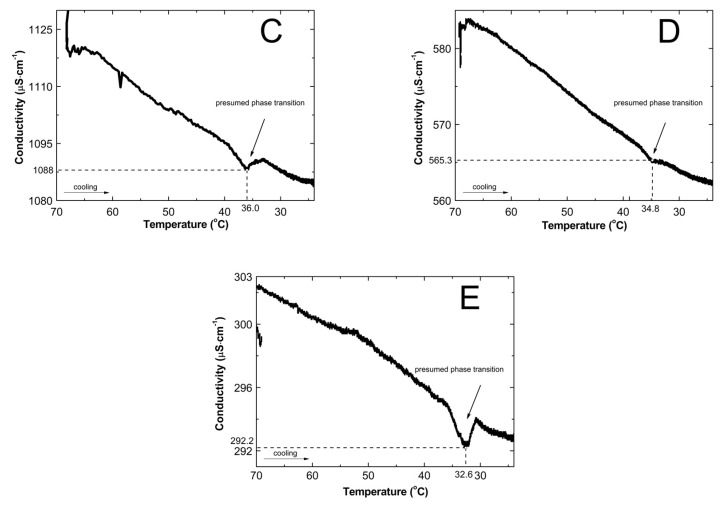
The conductivity as a function of temperature in the reaction mixtures of P1 (**A**), P2 (**B**), P3 (**C**), P4 (**D**), and P5 (**E**) during cooling of the system after the synthesis procedure.

**Figure 4:**
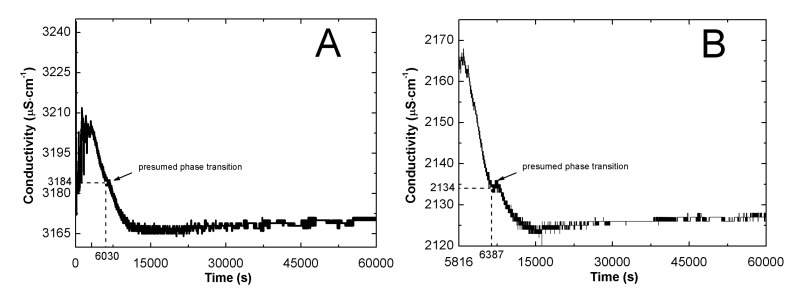

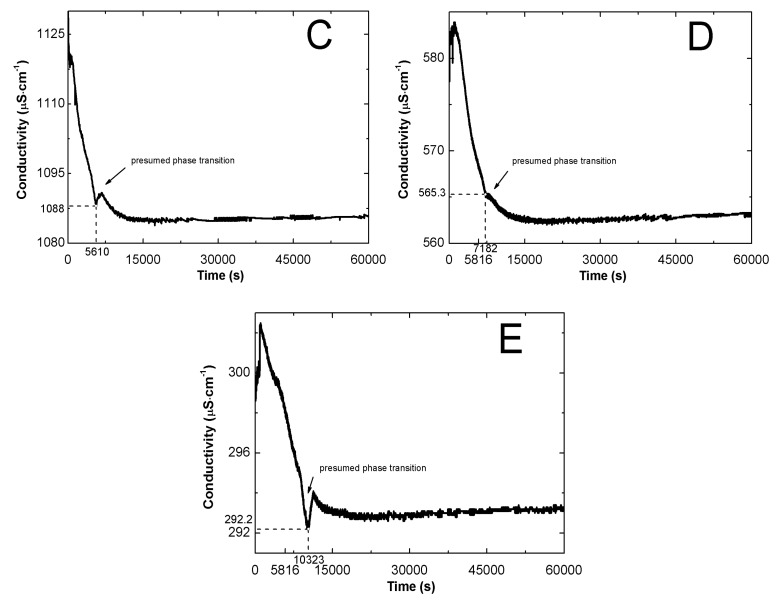
Conductivity as a function of time in the reaction mixtures of P1 (**A**), P2 (**B**), P3 (**C**), P4 (**D**), and P5 (**E**) during cooling of the system after the synthesis procedure.

The conductivity in function of temperature (cf. Figure 3) and of time (cf. Figure 4) was also measured during cooling of the systems.

The conductivity systematically decreased with the decrease of temperature. Moreover, at temperatures 37.5, 36.5, 36, 34.9, 32.6 °C for systems P1, P2, P3, P4, and P5, respectively, there were variations in the profile of conductivity. At the above-mentioned temperatures, the P1, P2, and P4 systems reached conductivity values of 3184, 2134, and 565.3 μS cm^−1^, respectively, which remained transitionally constant, and then gradually decreased to 3167 μS cm^−1^ for polymer P1, 2124 μS cm^−1^ for polymer P2, and 562.2 μS cm^−1^ for polymer P4. In P3 and P5 systems shortly before the expected phase transition, at temperatures 36.5 and 34.8 °C, respectively, the conductivity profile shifted from a slight to a rapid decrease. Further reduction of the temperature resulted in a sharp increase of conductivity from 1088 μS cm^−1^ in 36.0 °C to 1091 μS cm^−1^ in 33.0 °C, and from 292.2 μS cm^−1^ in 32.6 °C to 294.1 μS cm^−1^ in 30.7 °C for polymers P3, and P5, respectively. In the next stage the conductivity regularly decreased to 1085 and 293 μS cm^−1^ at 24 °C. Only the solutions of polymer P1 and P2 reached the plateau phase at temperatures 27.7 and 26.2 °C, respectively.

According to the data in Figure 4A–E, an almost exponential decrease of conductivity in time was observed. The plots had an initial large slope, but after 6030, 6387, 5610, 7182, and 10,323 seconds the continuity was disturbed, which was reflected in the small concavity on the graphs, with minimums at 3814, 2134, 1088, 563.3, 292.2 μS cm^−1^ for P1, P2, P3, P4, and P5, respectively. The deflection was visibly marked for polymeric systems P3 and P5. The presented aberrations were followed by regular exponential decay.

### 2.3. Attenuated Total Reflection Fourier Transform Infrared Spectroscopy Analysis (ATR-FTIR)

**Figure 5:**
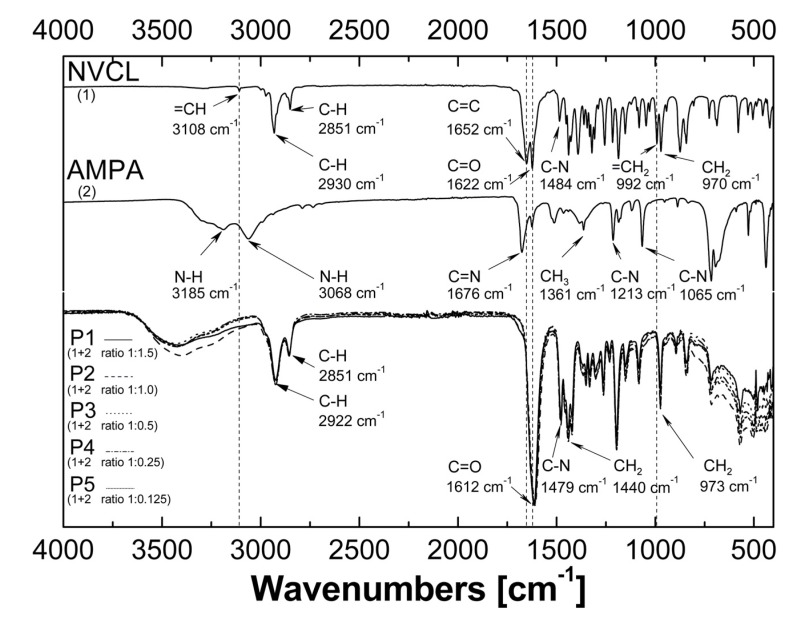
Fourier-transformed infrared spectroscopy with attenuated total reflectance (ATR–FTIR): spectra of monomers *N*-vinylcaprolactam (NVCL), initiator 2,2′-azobis(2-methylpropionamidine) dihydrochloride (AMPA), and synthesized polymers P1, P2, P3, P4, and P5.

The attenuated total reflection Fourier-transformed infrared spectroscopy (ATR–FTIR) spectra of *N*-vinylcaprolactam (NVCL), initiator 2,2′-azobis(2-methylpropionamidine) dihydrochloride (AMPA), and synthesized polymers P1, P2, P3, P4, and P5 are presented in Figure 5. The spectrum of NVCL exhibited characteristic bands at 1622 cm^−1^ due to carbonyl peak C=O, at 1652 cm^−1^ attributed to double bond C=C, at 3108 and 992 cm^−1^ corresponded to the =CH and =CH_2_ vinyl peaks, respectively, at 1484 cm^−1^ C–N stretching vibration, and at 970 cm^−1^ –CH_2_ twisting vibrations originating from the seven-membered ring [20], furthermore, at 2930 and 2851 cm^−1^ related to C–H stretching [15]. In the case of AMPA, the spectrum contained peaks in the range 3300–3000 cm^−1^ characteristic for the primary amines, estimated as asymmetric N–H stretch at 3185 cm^−1^ and symmetric N–H stretch at 3068 cm^−1^. The peak at 1676 cm^−1^ can be attributed to C=N stretching vibration. The positions at 1213 and 1065 cm^−1^ corresponded to C–N stretching vibrations. The peak at 1361 cm^−1^ was due to presence of methyl group –CH_3_. The spectra of polymers exhibited broad band at 3600–3000 cm^−1^ belonged to the O–H stretching. The peaks at 2922 and 2851 cm^−1^ corresponded to the aliphatic C–H stretching vibrations. The strong band at 1612 cm^−1^ can involve of bending vibration C=O. The peaks at 973, 1440, and 1479 cm^−1^ can be attributed to the twisting vibrations of –CH_2_, stretching vibration of –CH_2_, and CN bond, respectively [15].

### 2.4. Nuclear Magnetic Resonance Spectroscopy Analysis (^1^H NMR)

**Figure 6:**
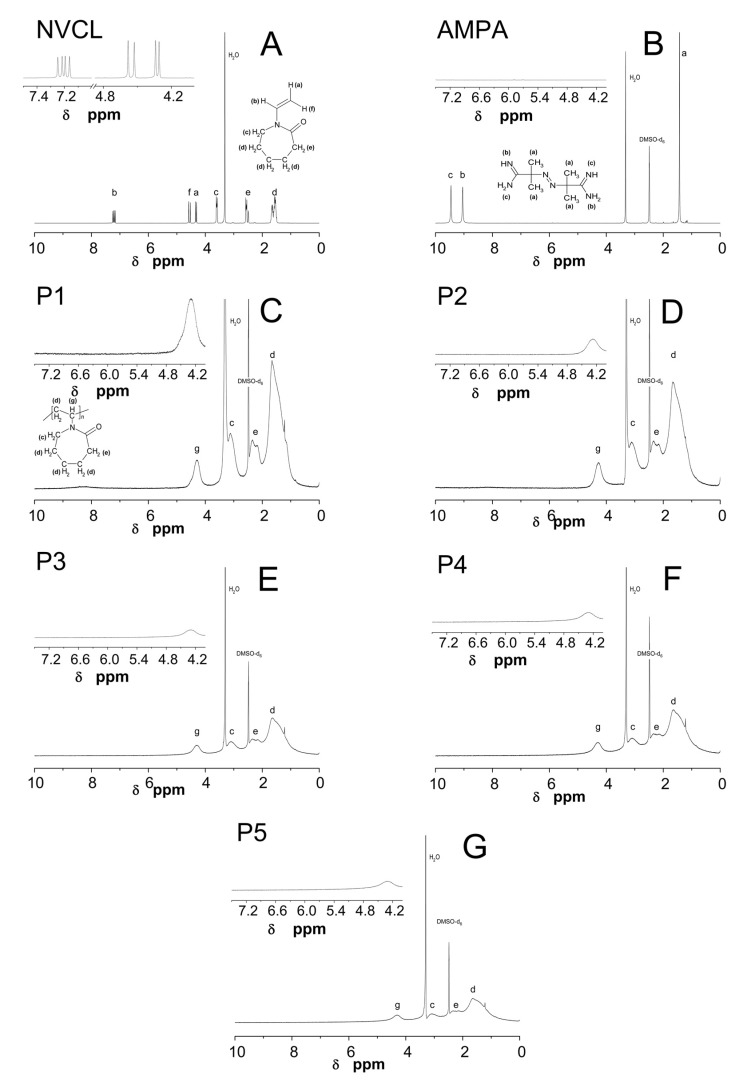
^1^H NMR spectra of monomer NVCL (**A**), initiator AMPA (**B**), and five synthesized polymers P1 (**C**); P2 (**D**); P3 (**E**); P4 (**F**); and P5 (**G**). The expanded areas in the ^1^H NMR spectra present the resonance range of the vinyl protons.

^1^H NMR spectra of monomer NVCL, initiator AMPA, and five synthesized polymers P1, P2, P3, P4, and P5 are given in Figure 6A–G. In the ^1^H-NMR spectrum of NVCL six groups of equivalent protons were marked in the spectrum as a, b, c, d, e, and f (cf. Figure 6A). The signals in the range δ = 4.27–4.36, δ = 4.48–4.66 and at δ = 7.17 originated from the protons a, f, and b of the vinyl group –CH=CH_2_ respectively. The other peaks with chemical shifts around δ = 3.61, δ = 2.58 and in the range δ = 1.38–1.76 corresponded to protons c, e, and d from groups –NCH_2_, –COH_2_, and –CH_2_ in the lactam ring, respectively. In the ^1^H-NMR spectrum of the initiator AMPA there are peaks from three groups of equivalent protons at δ = 9.46, δ = 9.06, and at δ = 1.44, designated in the figure as a, b, and c, associated to the protons from groups =N–H, –NH_2_, and –CH_3_, respectively. Figure 6C–G depict ^1^H NMR spectra of prepared polymers P1, P2, P3, P4, and P5. In all spectra of polymers, four peaks can be detected at the same chemical shifts around δ = 4.30, δ = 3.12, δ = 2.35, and δ = 1.65 belonging to –NCH, –NCH_2_, –COCH_2_, and –CH_2_. Identified peaks have been marked in the spectra as g, c, d, and e in the order of decreasing chemical shift. The same peaks on spectra of polymer P1, P2, P3, P4, and P5 had different intensities. The highest intensity exerted peaks on the spectrum of polymer P1 and the lowest on the spectrum of polymer P5. In the recorded ^1^H NMR spectra peaks at δ = 3.30 and δ = 2.48 corresponded to the H_2_O and the solvent, dimethyl sulfoxide-d_6_, respectively.

### 2.5. Hydrodynamic Diameter (HD)

**Figure 7:**
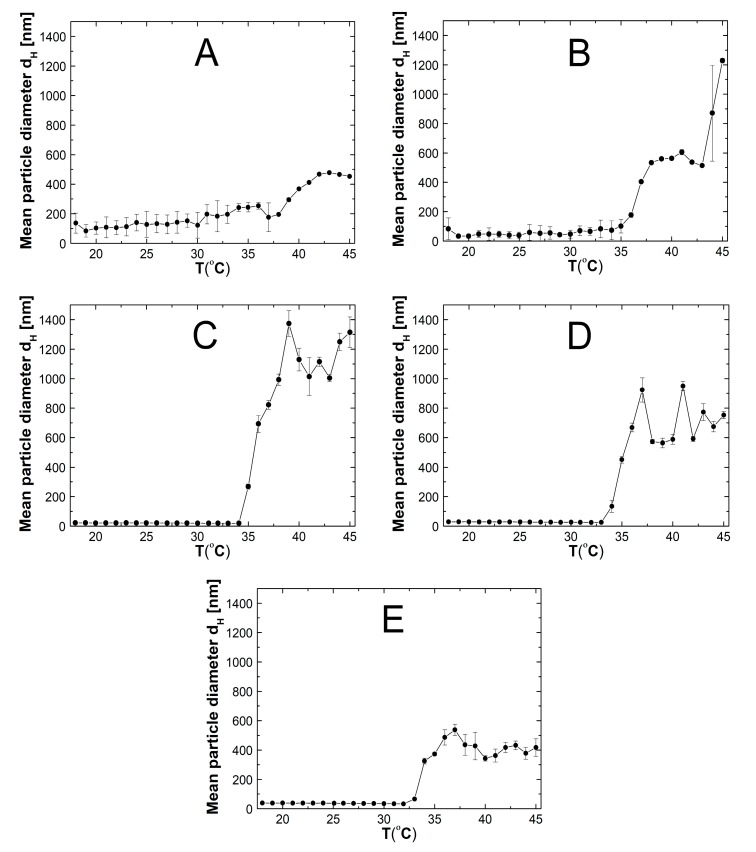
The effect of the temperature on the hydrodynamic diameter of the prepared particles P1 (**A**), P2 (**B**), P3 (**C**), P4 (**D**), and P5 (**E**).

Figure 7A–E presents the influence of temperature increase on the particle hydrodynamic diameters assessed in aqueous suspensions of synthesized polymers P1, P2, P3, P4, and P5. The d_H_ vs. temperature plots of assessed polymeric samples had similar change profiles with sharp increases reflecting the phase transformation temperature. Up to temperatures of 38, 35, 34, 33, and 32 °C for the polymers P1, P2, P3, P4, and P5, respectively, the hydrodynamic diameter remained stable or varied very slightly with insignificant deviations. Exceeding of the above-mentioned temperatures resulted in rapid increase of the hydrodynamic diameter, followed by a small increase.

The average value of the hydrodynamic diameter of the polymer P1 in the range 18–38 °C was 156.46 ± 49.58, whereas above 38 °C up to 43 °C the hydrodynamic diameter increased sharply from 192.45 to 477.28 nm. Then, as the temperature raised, subtle changes in the hydrodynamic diameter were observed; the average value of d_H_ in the temperature range of 43–45 °C was 464.93 ± 12.53 nm. In the case of polymer P2 (cf. Figure 7B), during the temperature rise from 18 to 35 °C, the size of the hydrodynamic diameter did not change significantly and the average value of the hydrodynamic diameter in this temperature range of was 56.96 ± 18.95 nm. Between temperatures 35 and 41 °C, the hydrodynamic diameter increased almost six times from 101.34 to 605.98 nm. Measurements at 42 and 43 °C showed a decrease in hydrodynamic diameter to 514.14 at 43 °C and the next two measurements showed a remarkable increase to 1228.90 nm at 45 °C. As illustrated in Figure 7C the hydrodynamic diameter of polymer P3 in the temperature range of 18–34 °C reached sizes from 19.04 to 22.11 nm. The average hydrodynamic diameter and standard error obtained for all measurements in this range were 20.09 ± 0.95 nm. From temperatures 35–39 °C the hydrodynamic diameter of the particles increased abruptly and achieved a value of 1373.80 nm. The fluctuations of changes in the hydrodynamic diameter occurring in the temperature range of 39–45 °C were greater than at lower temperatures 18–35 °C, but values of the hydrodynamic diameter that the particles achieved above the temperature 39 °C did not exceed 10% of the value of 1373.80 nm (at 39 °C). The curve mean particle diameter-temperature for polymer P4 showed that the size of particles in the temperature range of 18–33 °C remained constant with small standard deviations and the average hydrodynamic diameter was 27.60 ± 1.45. The characteristic, quick increase in hydrodynamic diameter from 134.24 to 923.50 nm occurred in the temperature range of 34–37 °C. Over the temperature 37 °C there was reduction in the hydrodynamic diameter to 588.32 nm at 40 °C next at 41 °C the hydrodynamic diameter increased to 949.80 nm and at 42 °C again decreased to 591.48 nm. Ultimately, at 45 °C, the particles reached a size of 752.12 nm. Figure 7E shows that with the increase in temperature from 18 to 45 °C, the hydrodynamic diameter of polymer P5 increased from 39.18 to 417.20 nm. Between 33 and 37 °C there was a sharp growth in the size of the particles from a value of 325.24 to 537.54 nm. The average value of the particles was 36.73 ± 1.89 and 401.80 ± 35.13 in the temperature range of 18–32 °C and 38–45 °C, respectively.

### 2.6. Polydispersity Index (PDI)

**Figure 8:**
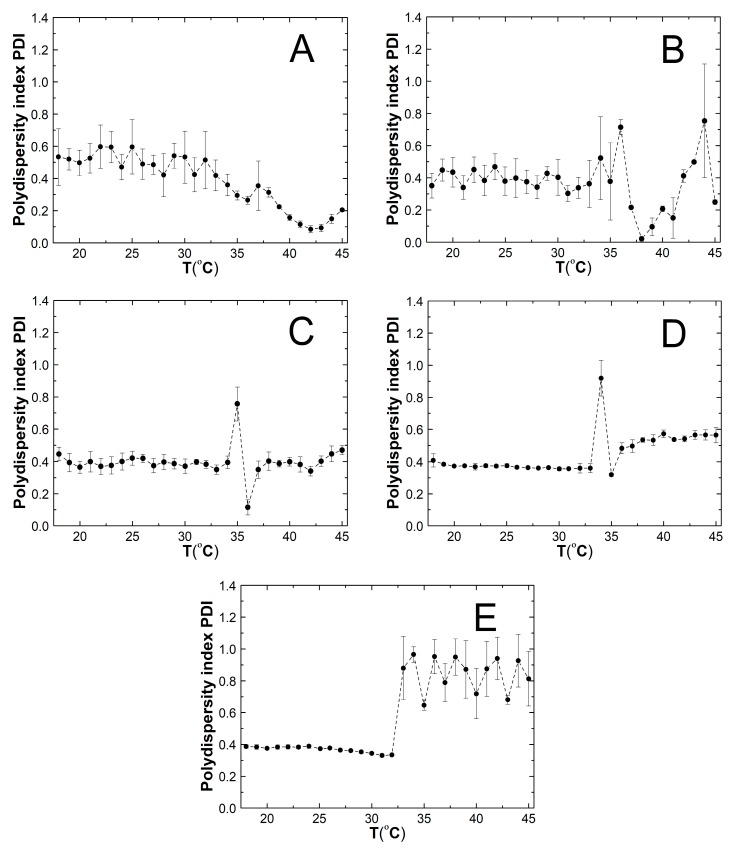
The effect of the temperature on the polydispersity index (PDI) of the prepared nanoparticles P1 (**A**), P2 (**B**), P3 (**C**), P4 (**D**), and P5 (**E**) obtained from dynamic light scattering (DLS) measurements.

Figure 8A–D shows the values of the polydispersity index (PDI) of synthesized polymeric nanoparticles as a function of temperature in the range 18–45 °C. In all cases similar, initial temperature trends were observed. In the beginning, the PDI values remained at a constant level up to certain temperature: 32 °C for P1, 35 °C for P2, 34 °C for P3, 33 °C for P4, 32 °C for P5, and were on average 0.52, 0.39, 0.39, 0.37, and 0.37, respectively. It should be noted that the PDI values for P1 and P2 polymers, in the indicated temperature range, were affected with large values of the standard error constituting approximately 20% and 25% of the measured value, respectively. Then, as the temperature increased, the PDI values distinctively decreased. In the case of polymer P1 (cf. Figure 8A) there was a gradual decrease in the PDI value to 0.08 at 42 °C, followed by an increase to 0.2 at 45 °C. Changes in the PDI for polymer P2 occurred similarly as for polymer P1 (cf. Figure 8B). The PDI value increased more than 1.5 times from 0.38 at 35 °C to 0.71 at 36 °C and next dropped to 0.02 at 38 °C and again increased to 0.75 at 44 °C. An increase in temperature by one degree caused a decrease in the PDI value to 0.25. The profile of changes in PDI values in the area of temperature phase transition for P3 and P4 polymers was the same. As can be seen in the diagrams in Figure 8C,D the following changes occurred successively: a rapid increase in PDI value from 0.39 at 34 °C to 0.76 at 35 °C for polymer P3 and from 0.36 at 33 °C to 0.92 at 34 °C for polymer P4, then a sharp decrease in the PDI value to 0.12 at 36 °C for polymer P3 and to 0.32 at 35 °C for polymer P4, then a re-increasing PDI to 0.35 at 37 °C for polymer P3 and to 0.48 at 36 °C for polymer P4. In both cases, when the temperature rose to 45 °C, the PDI values remained, with slight fluctuations, at a constant level of about 0.40 and 0.54 for polymer P3 and P4, respectively. Figure 8E shows that significant and linear changes in PDI values for the P5 polymer from 0.33 to 0.97 occurred in a temperature range of 32–34 °C. In the temperature range of 35–45 °C the PDI value alternately increased and decreased within 0.65–0.95 and exhibited standard error values between 0.03 and 0.18.

### 2.7. Zeta Potential (ZP)

**Figure 9:**
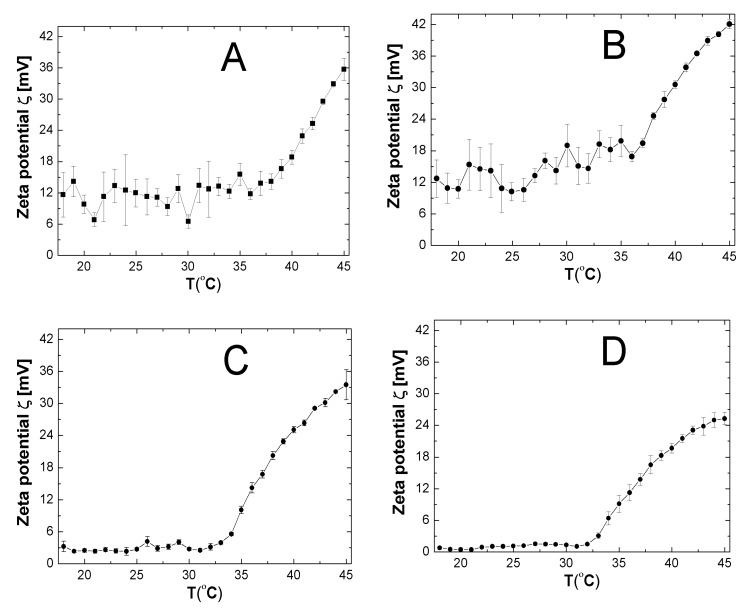

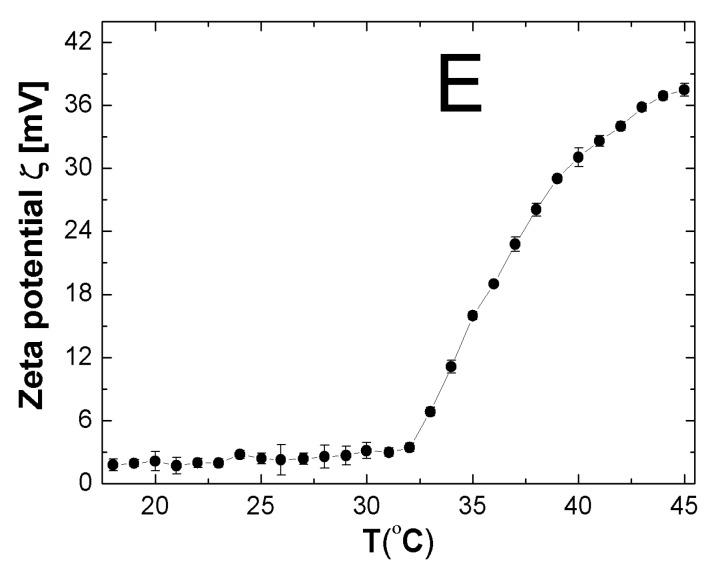
The effect of the temperature on the zeta potential (ZP) of the prepared nanoparticles P1 (**A**), P2 (**B**), P3 (**C**), P4 (**D**), and P5 (**E**) obtained from dynamic light scattering (DLS) measurements.

The influence of temperature on the zeta potential (ZP) of synthesized cationic polymers was investigated in the temperature range of 18–45 °C in unbuffered, aqueous solutions (Figure 9A–E). The pH values measured for all purified polymer solutions at ambient temperature ca. 25 °C were 7.45, 6.99, 7.19, 6.85, and 7.02 for polymers P1, P2, P3, P4, and P5, respectively. All tested polymers had positive values of ZP in the entire assessed temperature ranges. The similar pattern in the temperature trend of ZP for all studied polymers was observed, namely, in each case, two areas above and below the LCST can be distinguished. There were no significant differences in ZP values as the temperature increased to the polymer phase transition temperature, however, the polymers P1 and P3 exhibited greater fluctuation of changes in the value of zeta potential. After exceeding the lower critical temperature solution, the ZP value increased rapidly and linearly with growing temperature. The effect of temperature on the ZP of polymer P1 is presented in Figure 9A. In the temperature range of 18–36 °C, the zeta potential ranged within 6.52–15.56 mV. Above 36 °C, the ZP value increased rapidly reaching 35.70 mV at 45 °C. Figure 9B showed changes of ZP versus temperature for polymer P2. Between temperatures of 18 and 36 °C, the zeta potential values varied from 10.22 to 19.26 mV, then the zeta potential grew rapidly, reaching 42.08 mV at 45 °C. The temperature trend of ZP for polymer P3 is presented in Figure 9C. From temperatures 18–33 °C the value of the zeta potential varied from 2.31 to 4.15 and its average value was 2.94. Above 33 °C, the value of ZP increased from 5.57 to 33.50 at 45 °C. Zeta potential values for the P4 polymer in the temperature range of 18–32 °C did not exceed the value of 2 and its changes occurred in the range from 0.43 to 1.53 mV. However, in the temperature range of from 33 to 45 °C, the ZP values were constantly rising from 3.05 to 25.30 mV, cf. Figure 9D. As can be seen in Figure 9E, the zeta potential changes for the polymer P5 in the temperature range of from 18–31 °C were very gentle. Zeta potential in this temperature range of was between 1.72 and 3.14 mV. An increase in temperature from 32 to 45 °C caused more than a ten-fold increase in zeta potential from 3.44 to 37.48 mV.

### 2.8. Thermogravimetric Analysis (TGA)

**Figure 10:**
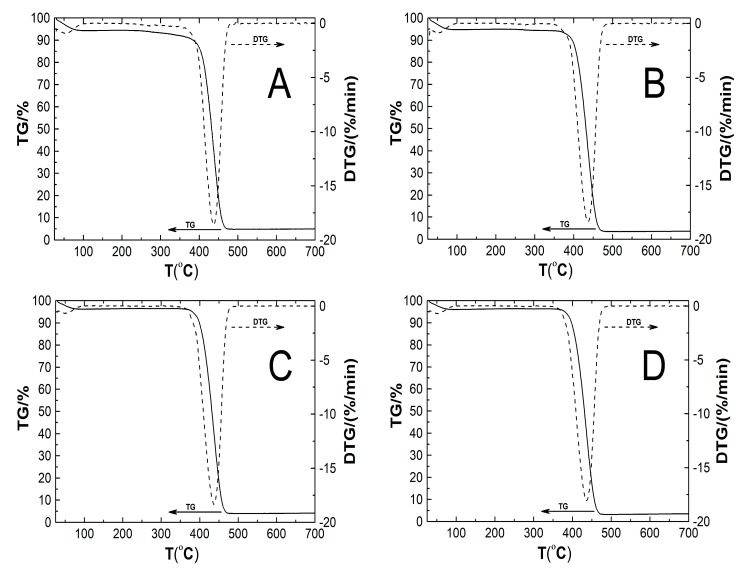

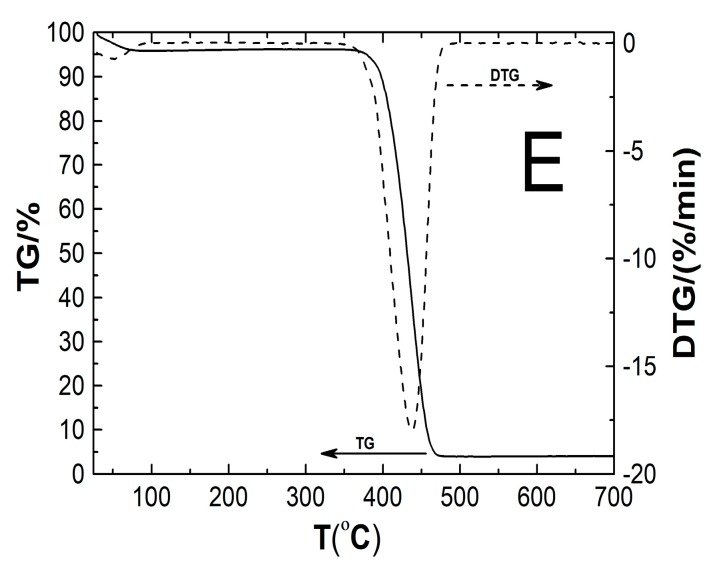
TG (solid line) and DTG (dash line) curves of weight loss (%) vs. temperature of polymers P1 (**A**), P5 (**B**), P3 (**C**), P4 (**D**), and P5 (**E**) obtained at heating rate of 10 °C min^−1^ in a nitrogen atmosphere at 50 mL min^−1^.

The characteristic TG and DTG curves of the thermal decomposition of nanospheric polymers P1, P2, P3, P4, and P5 obtained at a heating rate of 10 °C min^−1^ are shown in Figure 10A–E.

The TG plots represent the percentage mass loss of tested polymeric samples in the temperature range of from 29 to 800 °C. In all cases TG curves presented a similar course of thermal degradation. In the temperature range of 29–100 °C there was a gradual ca. 5% mass loss, then, up to 350 °C, no weight loss was observed. At temperatures above 350 °C there was a significant ca. 92% weight loss, finishing at about 495 °C. Then the mass of samples was stable until ca. 800 °C, and accounted for ca. 3% of the initial mass. In the DTG plots two peaks were always observed: one at about 52 °C and the second around 437 °C. The characteristic thermal decomposition parameters, e.g., extrapolated onset temperature of decomposition (*T*_Onset_), temperature of maximum weight loss rate (*T*_m_), extrapolated temperature at which the degradation process ended (*T*_Endset_), the temperature at which 0.5 wt% loss occurred (*T*_0.5wt%_), and amount of residue at for all samples determined by thermogravimetric analysis are summarized in Table 2.

### 2.9. Powder X-ray Diffraction Analysis (PXRD)

**Figure 11:**
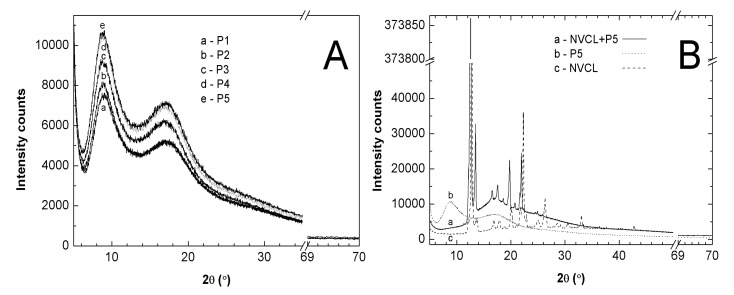
Powder X-ray Diffraction of powder samples: (**A**) five samples of synthesized polymers P1, P2, P3, P4, and P5, and of (**B**) polymer P5, monomer NVCL, mixture NVCL+P5.

The PXRD diffraction pattern of obtained nanospheric polymers P1, P2, P3, P4, and P5 in powder form are illustrated in Figure 11A. The diffractograms of all polymeric samples present two broad maxima at approximately the same reflection angles, the first at ~8° 2θ and the second at ~17° 2θ. The Figure 11B presents the comparisons of three PXRD diffractograms recorded for commercial monomer NVCL (dashed line), synthesized polymer P5 (dotted line), and a mixture of mechanically-bounded monomer NVCL and polymer P5 (solid line). The PXRD pattern of commercial monomer NVCL showed crystalline nature. The characteristic and strongest crystalline peaks of NVCL sample were detected at 2θ values of 12.75° and 22.29°. The diffractogram of polymer P5 showed that there was no crystalline peaks and two broad halos were observed at 2θ of 8° and 17°. PXRD patterns of mixtures, consisting of NVCL and P5, indicate the presence of both the crystalline and amorphous phases. The two peaks, characteristic for NVCL, become definitely more intense and were slightly shifted from 2θ = 22.29° to 2θ = 21.98° and from 2θ = 12.75° to 2θ = 12.55° positions. Moreover, at 2θ of 13.48° and 19.79°, there were shifted, but clearly visible, intense peaks which remained very weak in the monomer diffraction pattern. The comparison of the diffractograms of polymer P5 and the monomer-polymer mixture showed a complete disappearance of the large halo at 8° 2θ on the mixture diffractogram.

## 3. Discussion

### 3.1. Synthesis

**Figure 12:**
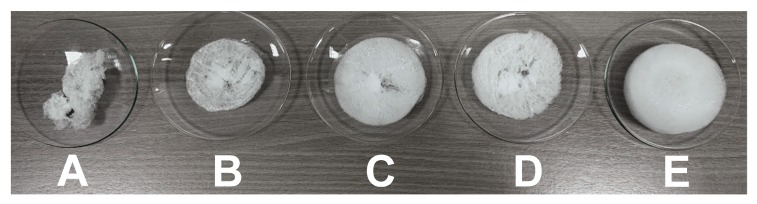
Freeze-dried samples of P1 (**A**), P2 (**B**), P3 (**C**), P4 (**D**), P5 (**E**) polymers.

As a result of the syntheses, five reversible water-soluble polymers P1, P2, P3, P4, and P5 were obtained. In each case, in the reaction mixtures heated to 70 °C after the addition of the monomer solution, the appearance of turbidity was observed, which slowly disappeared as the temperature was reduced. The turbidity/transparency effect arbitrarily confirmed that volume phase transition was occurred and the resulting polymer material was thermo-responsible. The lyophilization of purified aqueous polymer solutions resulted in a solid amorphous product resembling cotton wool and from the 100 mL of solution, by volume and by weight, the largest amount of solid product was obtained from the sample of polymer P5 and the smallest from polymer P1, cf. Figure 12.

### 3.2. Conductivity

The measurements of the change in the conductivity of the aqueous phase of reaction mixture may be a useful method for determining and characterizing polymerization steps during the free radical polymerization course [21,22]. The data from conductometric measurements may also be used to determine the kinetic parameters of the NVCL polymerization reaction. Due to the fact that radical reactions occur very quickly the precise and detailed registration, observation, and interpretation of conductivity changes at the beginning of the polymerization process, i.e., immediately after introducing the monomer solution into the reaction chamber, is very important. The experimental data presented in the Figure 2A–E allow to observe that the profile of conductivity changes in the first stage of polymerization, which can be attributed to the formation and increase in the number of growing oligoradicals, is significantly differ for the P3 polymer system. After the addition of the monomer to the reaction vessel, the conductivity values dropped in the case of polymers P1, P2, P4, and P5 very sharply, and for polymer P3, at first gently and then violently. It is clear that in the reaction system only at a specified monomer to initiator mole ratio of 1:0.5 created a factor that reduces the initial rate of oligoradical formation. The changing a certain system conditions, such as temperature, pressure, and the presence of other compounds, can cause the molecules in solutions to form spatially-organized structures called aggregates (micelles, lamels). The resulting structures can have different physical properties, e.g., surface tension, solubility, and viscosity compared to individual ones and so-called discontinuity occurs in the physical properties of the solution.

The increase in oligoradicals concentration is only a temporary process, the next stage may be chain growth, agglomeration or capture of oligoradicals by existing colloidally stable particles. The renewed increase in conductivity in all P1, P2, P3, P4, and P5 reaction systems may be the result of the formation of spherical polymer particles and their automatic collapse due to the temperature conditions in which they reside (*T* = 70 °C, higher than LCST of PNVCL). The shrinking of particles may cause the ejection of charged chemical groups existing inside the sphere on the surface and increase their density. The above considerations are reflected in the observations of turbidity, which is visible at the beginning of the increase in conductivity value (cf. Figure 2A) or a few minutes after the start of the rise (cf. Figure 2B–E).

The lowering of conductivity values by reducing the temperature is a natural process. Moreover, the increase in polymer particle size due to swelling also contributes to a reduction in the conductivity of the solution. However, in our case all deviations from the continuity of conductivity changes are very interesting. They may indicate physical changes in the studied system, for example, swollen particles. The study of the dependence of the conductivity of aqueous solutions of P1, P2, P3, P4, and P5 polymers on time and temperature during the cooling of reaction systems allowed to determine the LCST for each polymer and the time after which the phase change took place. Analyzing the data in Figure 3A–E presented the changes of conductivity in function of temperature, the relationship between the initial concentration of initiator and temperature of phase transition can be seen: the lower the initiator concentration, the lower the LCST the system achieves. This could indicate that reducing the initiator concentration results in the formation of smaller polymer particles. In addition, it could also be a confirmation that the increase in the hydrodynamic diameter of the particles registered in DLS studies in P4 and P5 systems is the result of aggregation. In the case of changes of conductivity in function of time in reaction mixtures, one can see that the time after which the phase transition occurs increases from P1 to P2, then decreases for P3 and increases again for P4 and P5 polymeric system. This may mean that the lower the initial concentration of the initiator, with the exception of the P3 system, the more time the system needs for spatial rearrangement to change from the collapsed to swollen form.

The following occurrence of a plateau may indicate that, in the polymeric systems, the colloidally stable polymer particles have been formed and there are no processes that increase or decrease the conductivity, hereby signaling that the polymerization process has ended.

### 3.3. ATR-FTIR

ATR-FTIR spectra of polymers synthesized under different amount of initiator are identical, the absorption bands from polymers P1, P2, P3, P4, and P5 overlap. Therefore, given the fact that for each compound a unique spectrum is created, one can be sure that the same substance was formed during the five syntheses performed. A comparison of the spectra of substrates and products allowed the determination if the polymerization occurred. The analysis of the spectrum of the monomer and spectra of the obtained polymers shows that the peaks at 3108, 1652, 992 cm^−1^ characteristic for the C=C bond disappeared in the spectra of polymers. These is an evident confirmation that polymerization process of NVCL occurs. Additional proof NVCL polymerization is increase the intensity of peaks related to C–H (2922, 2851 cm^−1^) and C–N (1479 cm^−1^) stretching vibration from groups in polymer chain. A slight change in the position of the peaks responsible for the presence of CO, CN and CH stretching vibrations from 1622 to 1612 cm^−1^, from 1484 to 1479 cm^−1^, and from 2930 to 2922 cm^−1^, respectively, on the spectra of polymers relative to the monomer spectrum may also indicate the occurrence of polymerization. The shifts may due to the conformational changes in the polymeric spheres within the side groups and interactions between atoms have changed. The broad band appearing in the range 3700–3000 cm^−1^ on the spectra of polymers indicates the presence of hydrogen bonds in the obtained products. The peaks at 970 and 973 cm^−1^ in the monomer spectrum and polymer spectrum respectively are related to the –CH_2_ twisting vibrations in the caprolactam ring. Peaks that correspond to the –C–N, –C–C–C–O groups may appear at this wavelength. Signals from the –CO group can be excluded because carbon connected with oxygen by a single bond is not present in both the monomer and polymer molecule of *N*-vinylcaprolactam. The ring breathing or deformation vibration may occur in these region but vibrations from –C–C group are not interpretatively useful. Based on the data from the spectral database, it was found that in the infrared spectrum of cycloheptane at 950 cm^−1^ occurs a strong peak [23]. This confirm the peaks at 970 cm^−1^ in spectrum of NVCL and 973 cm^−1^ in spectrum of poly-NVCL mainly due to twisting vibrations of CH_2_ group in the seven-membered ring.

### 3.4. ^1^H NMR

The ^1^H NMR spectra of the NVCL, AMPA and five polymers P1, P2, P3, P4, and P5 in DMSO-d_6_ solution were measured as additional confirmation that the polymerization process was successful and to support the structure and composition of prepared polymers. Analyzing the ^1^H-NMR spectrum of NVCL the peaks originate from the protons of vinyl group were found in the range δ = 4.27–7.17 ppm (cf. Figure 6A). The strong chemical shift of the signal from the proton of the vinyl group, marked on the obtained polymers spectra as b, towards higher ppm values results from proton location in close proximity to the group –N–C=O. As an effect of comparison the ^1^H-NMR spectra of substrates (cf. Figure 6A,B) and products (cf. Figure 6C–G) it can be seen that on the spectra of the synthesized products there are no signals caused by the presence of vinyl group protons. This is the main evidence that, during the applied synthesis process, the *N*-vinylcaprolactam has undergone the polymerization reaction. In addition, on all polymer spectra, in place of a doublet derived from vinyl protons, marked in the spectra as a, a broad single peak appears at the chemical shift δ = 4.30 ppm identified as a signal belonged to the –NCH proton. Another confirmation of obtaining polymers is the decidedly increased width and intensity of the peaks derived from c, d, and e protons from the lactam ring in the polymers’ ^1^H-NMR spectra compared to the monomer spectrum. This may be due to the fact that during the synthesis of polymers the number of lactam rings in the polymer chain increases and, in addition, the lactam ring undergoes conformational changes, which causes modifications in the appearance of the obtained polymer spectra. Since the signal intensity understood as the area under the signals is directly proportional to the number of protons from which the signal originates, the signal intensity appearing on the spectra of polymers from the proton of the –CNH group was compared. The signal intensity of the peaks on the spectra decreases in order from polymer P1 to P3, and then from polymer P4 to P5 increases slightly. This may mean that the polymer chain length decreases as the amount of initiator used for synthesis decreases, but only until the monomer-to-initiator ratio obtained is 1:0.5 as a further reduction of the amount of initiator may cause a slight increase in chain length.

### 3.5. HD

Due to the DLS data it can be concluded that all synthesized polymers P1, P2, P3, P4, and P5 are temperature-sensitive. Interpreting the graphs in Figure 7A–E, the phase transition temperature was determined as 38 °C for P1, 35 °C for P2, 34 °C for P3, 33 °C for P4, and 32 °C for P5. The results show the trend: the smaller the amount of initiator used for the synthesis of polymers, the lower the temperature of the phase transition the product acquires. According to the literature data [24,25,26,27,28] after reaching the LCST value, the polymer particles should shrink and reduce their diameter. Data obtained from hydrodynamic diameter measurements indicate an increase in particle size above LCST, which is the reverse of the literature data. This may indicate that, as a result of the phase transition, small molecules have been formed, which show a great ability to combine into aggregates. Probably the read-out values of the hydrodynamic diameter above the LCST apply to aggregates, not to the size of a single particle. Aggregation occurs rapidly up to a certain point and then the changes of read-out values of the hydrodynamic diameter take place mildly. Theoretically, high perturbations, including initiator concentration, may result in rapid phase transitions and, hence, larger aggregates of the condensed phase, as it was presented by Eslami et al. [29,30]. This may indicate that after the system has reached a certain temperature, aggregation is probably limited. The increase in the particle size above the LCST was also observed in our previous studies [21,22] and was explained based on DLVO theory [31].

Based on DLS particle size studies, it can be concluded that the synthesized polymers P2–P5 are of the nano size. Whereas the obtained particles of polymer P1 have a hydrodynamic diameter slightly above of 100 nm, they do not meet the criteria to be classified as nanoparticles, although their sizes are not large and do not exceed 150 nm up to 28 °C. A relatively short nucleation period of the initiator, and the half-life of AMPA equal ca. 290 min in 70 °C, can suggest that the end product of the synthesis will be small in size [32,33] but this is not the only factor determining the size of the particles. In an aqueous environment, both substrates and products may undergo hydrolysis, coagulation, aggregation, secondary reactions, and transfer reactions. The order and speed of individual processes also have a decisive impact. Finally, the process of forming polymer particles is governed by the interaction of all components in the reaction mixture.

Analyzing the data obtained from the measurements of the hydrodynamic diameter of polymers P1, P2, P3, P4, and P5 clearly shows that the initial concentration of the initiator affects the dimension of the hydrodynamic diameter of the synthesized particles, however, this is not a linear relationship.

**Figure 13:**
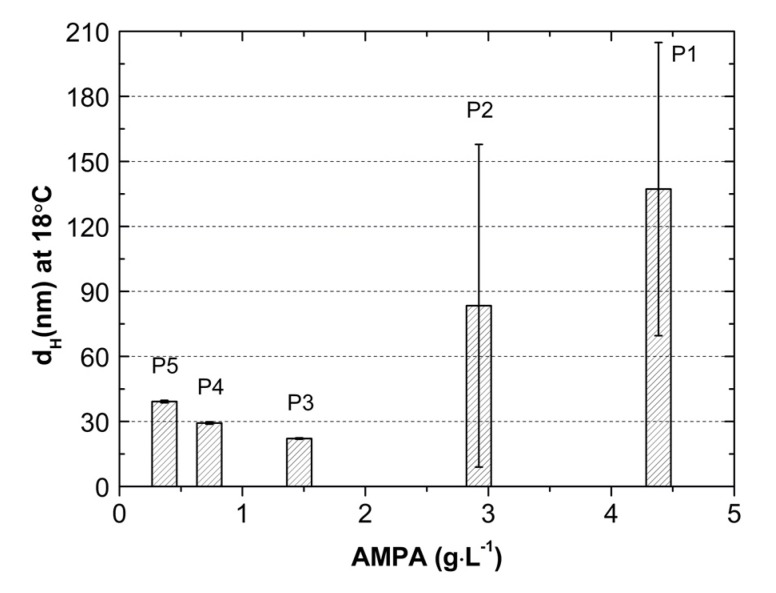
Particles sizes of prepared NVCL polymers P1, P2, P3, P4, and P5 as a function of the concentration of cationic initiator 2,2’-azobis[2-methylpropionamidine] dihydrochloride (AMPA).

Decreasing the concentration of initiator successively in the synthesis of polymers P1, P2, and P3 reduces the size of the hydrodynamic diameter of the final product. Further reducing the amount of initiator sequentially in the synthesis of P4 and P5 polymers results in an increase in particle size, cf. graph in the Figure 13. The particles prepared at higher concentrations of AMPA are subjected to a very large standard error which may suggest that they are less reliable. However, it should be remembered that the given hydrodynamic diameter is the average of five measuring cycles performed at one temperature in these case 18 °C. Therefore, large standard deviations may indicate that signals from both very large and very small particles were recorded during the measurement. In addition, supplementary and verification measurements were made of the sample from new synthesis, obtaining similar results, which is automatically a confirmation of repeatability of the synthesis and measurement method.

The observed behavior was completely different from what we obtained in our previous work in which the effect of the anionic initiator concentration on the particle size was studied [21]. It was found that the type and concentration of the initiator plays an important role in the size of the polymer particles. The similar effect, as in the present work, of reducing the particle size along with decreasing the initiator concentration was also observed by other researchers [32,33,34,35]. This effect was explained by the fact that the increased concentration of the initiator causes the formation of a large number of oligoradicals, which then combine into oligomers and next associate and coagulate to form larger particles. The particles grew until they were of colloidal stable size. Therefore, in continuous phase large particles, oligomers, and mature nuclei exist together. Probably, it was recorded in the measurements of the hydrodynamic diameter of the P5 and P4 polymers and resulted in a large standard error. Interestingly, when using, in the present work, an initial initiator concentration of less than 1.46 g L^−1^, the reverse effect was recorded, i.e., an increase in the hydrodynamic diameter of the particle polymers. Perhaps, as a result of the synthesis with a reduced amount of initiator, small particles were formed but were subject to aggregation and, as a consequence, the sizes of the formed aggregates was recorded and not size of individual particles. Anyway, we estimated the suitable concentration of the initiator under the synthesis conditions that allows the synthesis of the smallest, stable polymeric nanoparticles. Using an initiator at a concentration greater or less than the specified critical concentration results in obtaining particles with a larger hydrodynamic diameter.

### 3.6. PDI

Changes in the PDI values of synthesized polymers with increasing the temperature are given in Figure 8A–E. The obtained data indicates that at temperatures below the estimated LCTS the numerical value of PDI (which reflected size variability, i.e., polydispersity) of all polymers P1, P2, P3, P4, and P5 is quite low and did not extend to a value of 0.6. Moreover, the PDI values of the P1 and P2 polymers are higher and with larger standard errors (of about 20% and 25%, respectively) than in the case of the P3, P4, and P5 polymers. This may indicate that the application in polymerization of an initiator with a molar ratio of 1:1.5 and 1:1 of the monomer leads to the formation of particles with a broader distribution and greater polydispersity, what are reflected in the measurements of the hydrodynamic diameter. Above the phase transition temperature, only in the case of the P3 polymer did the polydispersity not change, and in the case of the P4 polymer it increased slightly and remaining at one level with growing of the temperature. These results point up that the particles of polymer P3 and polymer P4 were uniform and highly stable throughout the whole temperature range of used in the measurements. The values of the polydispersity index for polymers P1, P2, and P5, after exceeding the characteristic phase transformation point, changed rapidly. The polydispersion of the P1 and P2 polymeric particles lowered and then increased when the temperature rose. In addition, the PDI values for the P2 polymer are affected by a large standard error. Based on these results, one can conclude about continuous changes in particle size and instability in the P1 and P2 polymer system. In contrast, PDI values of the P5 polymeric nanoparticles increase to 1.0 and then alternately rose up and dropped on average by 0.2 units, as the temperature became greater. This proves the high polydispersity of the sample with multiple particle size populations. It is recognized that the particle size and particle distribution are important factors in determining product stability, but there are no specific criteria for accepted PDI values in various applications [36]. It can be considered that, in P3 and P4 polymers of PDI, about 0.4 can be deemed acceptable in drug delivery applications.

### 3.7. ZP

The zeta potential of the aqueous solutions of polymers P1, P2, P3, P4, and P5 was measured as a function of increasing temperature. The measurements recorded that the charge on the surface of polymeric particles was positive in the entire tested temperature range. The positive charge originates from the amine groups of the cationic initiator AMPA. In addition, in all cases the regular rise in zeta potential after the phase transition was observed, cf. Figure 9A–E. Continuously heating of an aqueous solutions of the P1–P5 polymers from 18 °C until their critical temperature did not affect the ZP and its values remain almost unchanged. Moreover, the use of an initiator in a ratio of 1:1.5 and 1:1.0 to the monomer, as is the case with polymer P1 and P2, respectively, leads to obtaining the higher positive surface charges than with polymers P3, P4, and P5. From this fact we can infer that the polymeric particles of P1 and P2 are larger than P3, P4, and P5 particles and accumulated more charge on their surface. These finding are consistent with the data obtained from HD and PDI measurements. Regardless of the amount of initiator used in the synthesis, the thermal shrinkage of P1, P2, P3, P4, and P5 particles above the LCST caused an increase in the ZP value. This can be attributed to the additional screening of positively-charged initiator fragments causing the enhancement of the surface charge density. The results indicate that polymeric particles of P1, P2, P3, P4, and P5 become more stable at higher temperatures [37].

### 3.8. TG

Thermal stability of nanospheric polymers P1, P2, P3, P4, and P5 was characterized by thermogravimetry and derivative thermogravimetry (DTG) as illustrated in Figure 10A–E. The same trends of the TG curves for all polymeric samples indicates that they have the same pyrolysis behavior. The thermograms show that all tested polymers P1–P5 are thermally stable up to about 350 °C and thermal degradation occurs in two steps. The initial, insignificant, ca. 5% weight loss may be due to the release of water molecules adsorbed from the air on the polymers’ surface or absorbed in the inner polymer. The second weight loss is related to the main decomposition stage, which is caused by the breaking of the covalent bonds in the polymer chain, vaporization, and then burning the formed gases, e.g., hydrogen, carbon oxide.

### 3.9. PXRD

The absence of sharp peaks on the all polymers’ patterns indicates that the obtained products are not crystalline and generally exhibit amorphousness. Upon decreasing the initiator content, there was a gradual and progressive increase of the peaks’ intensities, which may be due to the effect of the amount of initiator on the molecular structure of the polymer. The obtained results of the crystalline form of commercial NVCL correspond very well with the data from the literature [15]. The basic difference between the diffractogram of the monomer and the diffractogram of the NVCL-P5 mixture is the greater intensity and slight shift of the peaks of the crystalline form on the mixture PXRD pattern.

## 4. Materials and Methods

### 4.1. Materials

*N*-vinylcaprolactam (NVCL, 98%, Sigma-Aldrich Chemical, St. Louis, MO, USA) and 2,2′-azobis(2-methylpropionamidine) dihydrochloride (AMP, 97%, Sigma-Aldrich, Sternheim, Germany) were obtained from commercial and industrial suppliers and were used without further purification. NMR solvent—dimethyl sulfoxide-d_6_ (DMSO-d_6_, 99.96 at% D containing 0.03% tetramethyl silane-TMS) was purchased from Sigma-Aldrich CHEMIE GmbH, Sternheim, Germany. Dialysis tubing cellulose membrane (MWCO 12,000–14,000 Da) was received from Sigma-Aldrich CHEMIE GmbH, Sternheim, Germany. Deionized water fulfilled requirements of PN-EN ISO 3696:1999 for analytical laboratories; it was filtered in an HLP 20 device equipped with microfiltration capsule 0.22 μm (Hydrolab, Straszyn, Poland) and was applied in all following procedures.

### 4.2. Synthesis

Five derivatives of N-vinylcaprolactam, (P1, P2, P3, P4, and P5) were synthesized via the surfactant free precipitation polymerization (SFPP). First, the 2000-mL four-necked round bottom flask reaction vessel was filled with 900 mL deionized water, which is a solvent for all reactants, and heated to 70 °C (~0.09 μS cm^−1^, *T* = 25 °C). The reaction set was equipped in reflux condenser, temperature probe, conductivity cell (*K* = 1 cm^−1^), and magnetic stirrer (250 rpm). After nitrogen bubbling for ca. 30 min, an appropriate amount of the dry sample of free radical cationic initiator—AMPA, was introduced to the reaction vessel. Next, after ca. 10 min, the monomer (NVCL) dissolved in 100 mL of deionized water at room temperature with stirring in a beaker was added. The total volume of the reaction mixture was 1000 mL. The reaction was carried out over 6hours in with respective mixing and continuous nitrogen flow. The temperature of reaction mixture was kept constant of 70 ± 1 °C throughout the course of the reaction. Table 2 presents the different formulations for the performed polymerizations reactions. After polymerization, the reaction mixture was allowed to cool to room temperature and purified by dialysis against distilled water at room temperature. The sample of the reaction mixture (ca. 170 mL) was introduced to dialysis tubing cellulose membrane and placed in a 2000 mL cylinder filled with distilled water. During dialysis the water was stirred and changed every 24 h. The process of purification was finished when the conductivity of distilled water was about 1.5 µS cm^−1^. On average, the purification procedure involved six 24-hour cycles for every sample. Cleaned aqueous solutions of polymeric product (100 mL) were frozen, and lyophilized by Alpha 1–2 LD (Martin Christ Freeze Dryers, Osterode am Harz, Germany) two times for 26 h, and stored dry.

### 4.3. Conductivity Measurements

The conductivity of the aqueous solution of the reaction system in the course of the synthesis and during cooling procedure was measured using a model CC-505 laboratory conductometer (Elmetron, Poland) equipped with an EC-60 conductivity sensor with a glass housing and constant cell of *K* = 1.0 ± 0.2 cm^−1^ (Elmetron, Poland) and temperature sensor Pt-1000A (0–100 ± 0.35 °C). Measuring accuracy of the device up to 19.999 mS cm^−1^ was ±0.1% and from 20.000 mS cm^−1^ was ±0.25%. The conductivity and temperature sensors were placed into the reaction vessel and remained in permanent contact with the reaction mixture. Conductivity measurements during synthesis were performed at 70 °C manually and, during the cooling procedure, with an automatic compensation of temperature.

### 4.4. Attenuated Total Reflection Fourier Transformed Infrared Spectroscopy Measurements (ATR-FTIR)

The transmission and multiple attenuated total reflection infrared spectra of lyophilized samples were measured using a Nicolet iS50 FT-IR spectrometer (Thermo Fisher Scientific, Madison, WI, USA) with DTGS (deuterated triglycinesulphate) detector. Each spectrum was the Fourier transformation of 32 scans per sample collected at a resolution of 4 cm^−1^ over the wavenumber ranging from 4000 to 400 cm^−1^. The background spectra using a blank ATR were recorded before prior to applying the sample to the crystal and was automatically subtracted from measured spectrum of sample. The sample was applied directly on the area of the monolithic diamond crystal cell and compressed using a pressure arm. Before each measurement ATR was cleaned using methanol-soaked tissue paper.

### 4.5. Nuclear Magnetic Resonance Spectroscopy Measurements (^1^H NMR)

The ^1^H NMR spectra were recorded in NMR solvent deuterated dimetylsulfoxide, (DMSO-d_6_, δ = 2.49), at 299.15 K using a Bruker spectrometer with the working frequency of 300 MHz (Bruker, Rheinstetten, Germany). Coupling constants (J) are in Hertz (Hz) and chemical shifts (δ) are expressed in ppm. Analyzed by NMR, samples were prepared by dissolving about 10 mg of each compound in 7 mL of DMSO-d_6_.

### 4.6. Hydrodynamic Diameter (HD) and Polydispersity Index (PDI) Measurements

The hydrodynamic diameters of the nanopolymers aqueous dispersion were measured on a Zetasizer Nano device from Malvern Instruments (Malvern Instruments, Malvern, UK) by the dynamic light scattering method (DLS). The Zetasizer system was equipped with standard-red He–Ne laser, output power of 4 mW, and wavelength of λ = 633 nm, as a light source. The light intensity was adjusted during the measurement sequence by automatically setting the attenuator for the laser beam. The scattered light was detected at an angle of 173°, the backscattering setting. The DLS measurements were carried out in a temperature-controlled cell over a temperature range of 18–45 °C in one degree steps using a polyacrylic disposable DTS-0012 cuvette (Malvern Instruments, Malvern, UK). The cuvette was filled with 1 mL of polymer solution, purified by dialysis and not diluted. The measurements rounds were carried out five times at each temperature to give an average hydrodynamic diameter and size distribution. The number of replicates conducted in one round was adapted automatically in the range of 10–100 measurements. At each new temperature samples were allowed to equilibrate for 120 s. The polydispersity index (PDI) was calculated as a parameter of the particle size distributions using Malvern Instrument software based on the Stokes–Einstein equation for spheres. The size and PDI parameters were calculated according to the definition in the ISO 13321:1996E and ISO 22412:2008 [38,39,40].

### 4.7. Zeta Potential (ZP) Measurements

The zeta potential (ZP) was measured by analyzing the electrophoretic mobility of synthesized polymers nanoparticles in aqueous dispersion. The measurements were carried out by using a Zetasizer Nano device from Malvern Instruments (Malvern Instruments, Malvern, UK) in type DTS-1070 U-shaped cuvettes (Malvern Instruments, Malvern, UK) and applying the Doppler effect (laser doppler velocimetry, LDV). ZP values were calculated from the Smoluchowski model approximation to Henry’s equation (f(Ka) = 1.5). The electrophoretic mobility was measured as a function of temperature in the range of 18–45 °C in one degree steps and with an equilibration time of 120 s. At each temperature measurement rounds were executed five times.

### 4.8. Thermogravimetric Analysis (TGA)

The thermal stability of the synthesized polymeric nanospheres was analyzed by using a TG 209 F1 Libra instrument with automatic sample changer (ASC), (Erich NETZSCH GmbH and Co. Holding KG, Selb, Germany). The measurements were carried out under non-isothermal heating conditions in a temperature range of 29–800 °C (heating rate 10 °C min^−1^) under an N_2_ atmosphere (with a flow rate of 50 mL min^−1^). The lyophilized amorphous samples form of approximately 5 mg were loaded into a corundum crucible and gently pressed. The samples were not grated beforehand. During the heating the weight loss of samples as a function of temperature and time was recorded automatically. To determine the mass change and DTG curves the Netzsch Proteus 7.1.0 analysis software (Selb, Germany) was used.

### 4.9. Powder X-ray Diffraction Analysis (PXRD)

The X-ray powder diffraction (PXRD) patterns were measured on an X-ray diffractometer on a Bruker D2 PHASER diffractometer (Bruker AXS, Karlsruhe, Germany) with a LynxEYE detector using Ni-filtered CuKα_1.2_ radiation (1.5418 Å). All samples were previously crushed in a mortar and then measured at 295 K in ambient atmosphere under constant conditions of 30 kV, 10 mA. The PXRD diffractograms were measured at 2θ, in the Bragg–Brentano (θ/2θ) horizontal geometry between 5° and 70° with 0.02° increments, and a recording time of 1.0 s/step. The variable rotation was 15 min^−1^, with a divergence slit 0.6 mm in size.

## 5. Conclusions

Surfactant-free precipitation polymerization in an aqueous environment at 70 °C degrees is an effective method of synthesis the polymer of N-vinylcaprolactam. In these studies we conducted five syntheses that differed only in the initial concentration of the initiator. As a result of the polymerization process five thermosensitive poly-NVCLs: P1, P2, P3, P4, and P5, strongly aggregating after exceeding the lower critical solubility temperature, were obtained. The hydrodynamic measurements of the particles confirmed that products were of nano-size. The conductometric measurements during synthesis allowed monitoring of the polymerization process. The particle size measurement versus temperature by the dynamic light scattering method was a suitable method to determine the phase transition temperature of thermally-sensitive poly-NVCL. Furthermore, the conductometric measurements of the reaction mixture after the synthesis and during the cooling can also be used to determine the value of LCST. The phase transition temperature was determined for the all synthesized polymers and it has been found that the changes of the initial concentration of the initiator caused changes in the LCST value. The ATR-FTIR and ^1^H NMR confirm that the polymerization reaction of NVCL has occurred. The polymer solutions were also characterized by measurements of ZP and the polydispersity index depending on temperature. The results of PDI measurements showed a relatively low degree of polydispersity for polymers P1, P2, P3, and P4 in the entire temperature range whereas, in the case of the P5 polymer, after initiating the phase transformation the polydispersity index was close to unity. The recorded ZP values indicated the positive charge on the surface of the poly-*N*-vinylcaprolactam particles and stability at higher temperatures. The solid products of synthesis were also subjected to PXRD and DSC tests and as a result the amorphity of the poly-NVCL was confirmed and the characteristic thermal decomposition parameters were determined. Purified by dialysis, aqueous suspensions of the obtained polymers, have a pH in the range of 7.43–6.85, which makes them suitable for applications as drug carriers.

## Figures and Tables

**Table 1:** Compositions of P1, P2, P3, P4, and P5 nanoparticles.

Type of Polymer Nanoparticles System	Monomer (mol)	Cationic Initiator (mol)
	NVCL	AMPA
P1	2.184 × 10^−2^	16.165 × 10^−3^
P2	2.155 × 10^−2^	10.777 × 10^−3^
P3	2.187 × 10^−2^	5.391 × 10^−3^
P4	2.160 × 10^−2^	2.695 × 10^−3^
P5	2.163 × 10^−2^	1.347 × 10^−3^

**Table 2:** The characteristic thermal degradation parameters of P1, P2, P3, P4, and P5.

Type of Polymer Nanoparticle System	*T*_m1_ (°C)	Rate of Mass Loss 1 (% min^−1^)	*T*_m2_ (°C)	Rate of Mass Loss 2 (% min^−1^)	*T*_Onset_ (°C)	*T*_Endset_ (°C)	Res (%)	*T*_0.5wt%_ (°C)
P1	51.5	0.92	437.7	18.63	410.1	457.2	5.03	29.7
P2	52.2	0.90	437.3	18.41	408.8	457.1	3.68	29.5
P3	48.1	0.68	436.5	18.36	404.8	457.1	4.22	30.2
P4	53.7	0.71	436.0	18.06	404.9	456.9	3.62	30.4
P5	52.4	0.75	437.6	17.99	402.9	457.1	4.11	29.8
